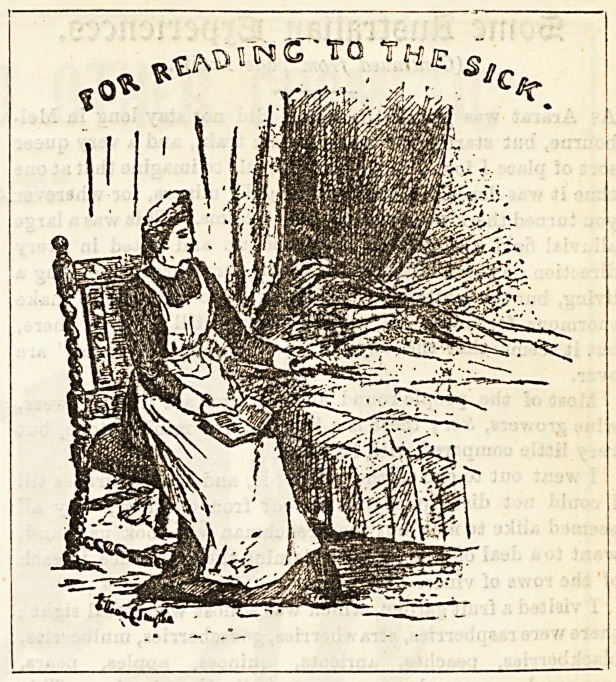# The Hospital Nursing Supplement

**Published:** 1892-11-12

**Authors:** 


### "The Hospital" Nursing Mirror.

Being the Extra Nursing Supplement of "The Hospital" Newspaper.

[Contributions for this Supplement should be addressed to the Editor, The Hospital, 140, Strand, London, W.C., and should have the word "Nursing" plainly written in left-hand top corner of the envelope.]

### En Passant.

THE R.B.N.A.—A meeting was held at the Mansion House, on Monday last, of the Special Committee on the Nursing of Cholera appointed by this Association. The Lord Mayor presided, and H.R.H. Princess Christian was present. The attendance was surprisingly small. Dr. Bezley Thorne (Hon. Secretary) said that the committee had organized a roll of nurses to serve in a cholera epidemic, and had made arrangements to bind nurses in their engagements to nurse, and to follow the instructions of those under whom they might be placed. Later on it was resolved, on the motion of Dr. Shirley Murphy, to institute a roll of higher grade nurses, capable of undertaking the duties of matron and district superintendent of nurses, in addition to nurses of lower grade.

FRIEDENHEIM.—The Princess Mary, Duchess of Teck, who was accompanied by the Princess May and Prince Adolphus, opened this Home for the Dying on Monday last, amongst a very large number of visitors. Too many tickets of admission had undoubtedly been given, and many present could neither see nor hear the proceedings, much to their disappointment. We were glad to find that the meeting resulted in several promises of funds to finish furnishing the home; but in order to set it going in good working order quite £3,500 are required. Sunnyside, which is the name of the new home, will accommodate forty to fifty patients, with a full staff of nurses and servants. The home is mainly for the poor but non-pauper class, and is essentially what its name describes it to be—"for the Dying"; and it is, with the exception of the Catholic Hospital of St. John and St. Elizabeth, in Great Ormond Street, the only refuge in London where peace can be found for those entering the valley of the shadow.

SHORT ITEMS.—We hear that Miss Clarisse Hunter, the Matron of the Foundling Hospital, Guildford Street, has resigned her post.—Nurse H. L. Wilson and Miss Binks have opened a private home for paying patients at "Eversley," West Kirby, Cheshire; the terms are from £2 2s. upwards. Miss Wilson is a well-known nurse, and we hope her home will succeed.—Nurses Redman and Lore have taken "Oakdene," London Road, St. Leonards-on-Sea, as a private nursing home. They will be able to accommodate about six invalids.—Nurse Bradley, who trained at Birmingham General Hospital, and who is nurse to the Bridport Nursing Society, has given much satisfaction to her committee. She paid 3,415 visits during the year and attended 175 patients.—We very gratefully acknowledge six shillings from the nurses at the Burton-on-Trent Nursing Institution s a contribution to our endowed bed for a sick nurse.—Miss Adams, who trained at the Brownlow Hill Hospital, Liverpool, has, we are pleased to hear, met with a warm welcome at Haputalle, Ceylon, where she went on the completion of her training to take charge of the Women's and Children's Hospital.—The Indian Nursing Service is to be increased very considerably.—Messrs. Sampson Low, Marston, and Co., have withdrawn from the publication of Miss Marsden's book "On Sledge and Horseback to Outcast Siberian Lepers."—Mr. Richard Benzon, of Englefield House, has given £500 to the purchase fund of the Helena Nursing Home, at Reading.

WINDSOR NURSES.—On November 3rd, the Princess Christian opened the new home which has been established at Windsor for the trained nurses of the district. The Dean of Windsor opened the proceedings with a religious service, after which the report was read, which referred to the affiliation of the society with the Q V.J.N.I., and to the good and efficient work it was undertaking among the poor. Princess Christian afterwards presented a gold badge to the Lady Superintendent, Miss Simpson, and silver badges to the five nurses.

THE ENDOWED BED FOR A SICK NURSE.—Once more we are obliged to come to our readers and ask them to do their best to help us maintain the free bed, so willingly subscribed for last year, during its second year of existence. We can ask for the money with comfort in the reflection that the bed is needed, and that the nurses who used it during this present year were restored to health, and enabled to go back to their work rested and happy. In the course of the next week we hope all subscribers will receive a subscription list, and we shall be very grateful for any additions to the fund, however small, all of which will be acknowledged in these columns. The bed, as most of our readers know, is at the Brassey Holiday Home, Ventnor, Isle of Wight, and a lovelier spot in which to rest it would be very hard to imagine. Sister Frost and Miss Holditch take much interest in the endowed bed, and have been uniformly kind to all the nurses who have used it. We shall be very glad to receive the names of any nurses needing a rest after illness or overwork whose means do not permit them to take one; and as we find there has been some little misconception of the use of our bed, we take this opportunity of saying that we do not want nurses to be really ILL before applying to us, but we hope in the coming year to keep many a poor nurse from such a catastrophe.

CHRISTMAS PARCELS AND COMPETITIONS.—We very gratefully acknowledge a parcel, containing six infants' vests, a comforter, two knitted neckties, one child's knitted petticoat, and a variety of other pretty and useful things sent to us for distribution in the London hospitals at Christmas by the Brassey Holiday Home; also two most excellent knitted shoulder shawls from Miss Lockyer, at Marlborough College, and four very well-made flannel petticoats from E. and R. S. In answer to correspondents the parcels must reach 140, Strand, not later than December 12th, and should be addressed to "Nursing," care of the Editor. We shall be glad if all senders will kindly write their names and addresses clearly. The prizes will be given in either books or money as the winners choose. (1) For the best pair of socks knitted by a nurse, 5s.; (2) for the best pair of socks knitted by any Hospital reader, 5s.; (3) for the best made flannel shirt, 10s.; (4) for the best made woman's blouse, 10s.; (5) for the best made flannel petticoat, 10s.; (6) for the best made and best shaped dressing-gown for an invalid, cut out and made by a nurse, 20s. It will be seen that Nos. 1 and 6 are reserved for nurses. Flannelette is cheap, and light, and warm, and would therefore form the best material for the dressing-gown. In judging, four marks are given for workmanship, four for shape, and two for general appearance; therefore it is not wise to spend time on elaborate trimmings. Long seams may be done by machine. If our readers will ask their friends to add something to our Christmas parcels, they will earn the gratitude of many a hospital worker. We shall be glad to distribute any clothing or presents of any sort besides those sent in for the competitions. To save confusion, will senders kindly mark such parcels "Not for Competition."

### Lectures for Asylum Attendants.

By William Harding, M.B.

### VII.—MANAGEMENT OF PATIENTS.

Did you ever reflect what an awful thing it is for a person not altogether unconscious of her situation to be brought to an asylum? The public are not yet fully educated up to looking upon such an institution as a hospital. There is still a lingering inclination to consider it a species of penitentiary, and the idea of entering it as a patient causes a shudder of horror to run through most people. Indeed, in the case of some melancholics the dread of the asylum has played a part in deepening the depression into which they have fallen. It is all essential that an endeavour should be made on the lunatic's first arrival at the asylum to teach her that her dark forebodings were groundless, and that she has arrived not amongst jailers, but friends.

You all know the influence of first impressions. Even on your first arrival as a nurse in an asylum you can recall how firm a hold your first ideas of the place took upon you. You were keenly sensitive to the nature of the greetings you received, and even to the tone of your companions' conversation. One who has been a patient in an ordinary hospital will tell you how helpful to him on his arrival was a cheering word and pleasant smile. How much more then is that friendly welcome essential in the case of the lunatic, whose future is often enshrouded with gloomy thoughts and dreadful apprehensions. Even if we considered the matter merely from the standpoint of convenience, without any higher motive, we would come to the conclusion that it is to our advantage to calm the patient's fears and allay her suspicions. If she once begins to look upon the nurse as her friend the latter will find her labours and anxieties lightened. There will be less danger of trouble with regard to food, &c., and the new-comer will more rapidly settle down amid her unusual surroundings. We often see a patient who has been difficult to manage outside calm down at once, and give little trouble when she finds herself amongst those who, while kind and sympathetic, are yet neither irritable, fussy, nor afraid. Few, unless quite demented, are utterly lost to outside influences. A smile or a friendly tone of the voice will frequently soothe and prevent an outburst of violence. The very way in which a patient's clothes were removed may be keenly remembered by her. Even if they have to be taken off against her will, this should always be done with every sign of good will.

It might be well to mention here some points which should not be overlooked on the admission of a new case. The doctor will of course examine for himself, but the nurse should none the less know what to take notice of when undressing the patient. Bruises or injuries should be carefully noted, and for two reasons.

Firstly: In order that any necessary treatment may be carried out.

Secondly: In order that an accurate note should be made of their existence and character. Even in my experience attempts have been made to throw the responsibility for injuries received before admission into the asylum upon the officials of that institution. It is only fair to the nurses in charge of the patient that the bruises present before admission should be recognised. It is enough that she is called upon to give an account should any bruises be received afterwards.

The presence of any marks on the body should be observed at the same time. Careful attention should be given to ascertain whether the patient be ruptured, and in the case of males whether there be any stricture of the urethra. The condition of the abdomen if at all prominent should be seen to, as the fulness may be due to a distended bladder, or even to pregnancy. In puerperal cases, and in nursing women, the condition of the breasts should be noted. One point that is very important, but is sometimes overlooked, is to make certain that there are no false teeth. These should always be borne in mind, as in some cases it may be necessary to remove them. I have known accidents occur from neglect of this precaution. The condition of the hair as regards vermin, &c., will not be forgotten.

When we come to speak of the general management of a body of the insane we find that much depends upon the temperament of the individual who has them in charge. The post of charge nurse is a difficult one, and calls for the exercise of high qualities in her who would fill it successfully. A clear head, strict impartiality, a subordination of her own feelings to her duty, an ability to see things unbiassed by the personal element, tact—these are the qualities one would like to see in a model charge nurse. She must, if she wishes to make the best of things for the patients, for her subordinates, and for herself, exercise that useful quality, tact. She must try to steer clear of tender places; must be careful not to hurt the feelings of those around her, and must practise the art of putting disagreeable things in their most pleasant aspect. We cannot expect human beings to be perfect, but there are a few rules that the nurse should bear in mind.

1st. Try from the very beginning to get the patient to understand that you are sincere and in earnest in your desire for her welfare. This is not always easy. The suspicious mind of the lunatic is often disposed to look upon all about her as enemies, and the nurse will frequently find her efforts misunderstood, and her kindly advances repaid with insolence or even violence.

2nd. Never hurt a patient's feelings by letting her see that you have gained a victory over her when you have been compelled to insist upon her doing something which she did not wish to do. If any such difficulty should arise, the nurse should try to raise a bridge by which the patient may cross without injuring her self-love. In dealing with the insane, it is quite possible to stoop to conquer without any loss of dignity or self-respect. The hectoring, loud-voiced individual is not always the strongest, nor the one who exercises most influence over her fellows.

3rd. Never force yourself into a position in which you must either sacrifice your influence or do something you would much rather have left undone. Thus never, unless under very exceptional circumstances, tell a patient that "she shall" or "she must" do so and so. "I think you had better" is much more efficacious, and even if it has to be enforced, does not leave the same sting behind it. Always try to note the patient's mood and catch the favourable opportunity. In cases where there is likely to be any conflict of opinion, get the order repeated in the hearing of the patient. If she have any reason at all left, the fact that in insisting upon a thing being done you are merely doing your duty, will have an influence with her. Unless when acutely excited, it is generally possible to let such a patient see that it is easier and better to do the right thing than the wrong. There are, of course, many lunatics whose reasoning powers are so impaired that they are quite unable to form any judgment, but that is not the class of whom I am speaking. The utterly irrational are, even when very violent, easily borne with as compared with the impertinence and insults from a perverse reasoning lunatic. You will rapidly find out those individuals who are most easily managed by being made much of; those who prefer a more distant greeting, and those generally incurable, hopeless cases, who are best dealt with by being left alone so long as they fall in with the rules of the house, and do not interfere with their fellows. Occasionally cases will arise where physical force must be resorted to. If possible a nurse should never attempt single-handed to deal with an excited or violent lunatic, but should always call assistance, and for the following reasons:—

1st. A patient will often submit quietly when two nurses are present, who would fight and struggle against one.

2nd. There is less likelihood of either the patient or the nurse being injured.

3rd. The patient is not so likely to make false accusations of ill-usage when two are present. Those who have had dealings with the insane, and more especially with epileptics, will appreciate the force of this last reason.

When aggravated by a patient's wilful perverseness, the nurse will at times feel a disinclination to call for assistance. She will be disposed to look upon this as an expression of weakness or cowardice, and will want to manage the patient by herself. From every point of view this feeling is wrong, and must be resisted. The power of self-control must be exercised. In fact, if a nurse be without that quality she will not be likely to be an asylum nurse for any lengthened period. It says much for the nursing staffs that so much is patiently borne and uncomplainingly suffered by them. Violence, insults, and abuse from the patients are more easily endured than the suspicions and impertinences of some of the relatives of the insane. As a rule it is those individuals who behaved badly to the patient before admission who are most troublesome, and insolently suspicious of ill-usage afterwards.

*(To be continued.)*

### Everybody's Opinion.

### A NURSE'S CAREER.

"Angelica" writes: I was trained in the far north, and at the end of my time family troubles have compelled me to come southwards and give up my idea of going on the private staff of our hospital. Now the question is where to go and what to do? and as I feel it is a question which perplexes many, I should like the opinion of other nurses who have found themselves in like difficulty. I know one spot where I might get on, as I have a medical friend there, but it means uphill work and much anxiety. The institutes I have applied to are certainly not models of generosity. Twenty pounds a-year is not a handsome remuneration for a nurse's services, and it seems to me that very few of the institutes have considered the desirability of giving any percentage on earnings. The Co-operation is the loadstone of many nurses, it sounds fascinating and lucrative, but we can't all join that because they will not want us. I am sure that if the institutes would only be more co-operative (for want of a better word), and would let their nurses' interests be the institute's interests, we should hear less abuse of them than we do at present. The question is which is best—to start on one's own account, and run a short risk for perhaps a long success, or be content with the small wages of an institute and have no risk? Surely, wages should begin for thoroughly-trained private nurses at £30 and rise to £40, rather than commence at £20 and rise to £35 after years of hard work?

### LECTURES ON SICK NURSING AND DOMESTIC ECONOMY.

"Justice" writes: I feel, in justice to the nursing lectures now going on in some counties, that I must send a few lines to your valuable paper just to prove there are some amongst them well worth hearing, and not inconsistent with the cottagers' means or style of living, as both economical and sensible advice is given, and I fear, from a letter recently published, some few may be prejudiced by it, and so deterred from attending them when an opportunity offers. I have been to the course of six, given in a Berkshire village, and found them good (alike for single or married), most thorough, practical, and plain, and the knowledge gained would be useful from the cradle to the grave. Our lectures were much appreciated, and few, if any, missed one of the series. The nurse gave a careful and instructive discourse, and several of the cottagers gladly availed themselves of the opportunity of practically working out by themselves the nurse's instructions, under her supervision. The gentleness and forbearance due to young or old, and even the reverent attention to the departed, was not forgotten, and I can but advise all who are fortunate enough to have such lectures near to attend them. If of the same valuable nature as ours were, I feel sure that none will do so without having learnt some useful hint for themselves, or one that may benefit a poor suffering neighbour.

### The Registration of Nurses.

### THE R.B.N.A. SCHEME REJECTED BY THE LORDS' COMMITTEE.

On July 2nd last we stated: "The Report of the Lords' Committee on the Metropolitan Hospitals ably sums up the question of the registration of nurses, as proposed by the Royal British Nurses' Association, which they find will be no protection to the public, though it would tend to reduce all nurses to one common level." We then printed paragraphs 478 to 483 of the Lords' Report inclus[illegible], which elaborated and justified the foregoing statement.

At the annual meeting of the R.B.N.A., held at Brighton shortly afterwards, Dr. Bezley Thorne, one of the hon. secretaries of the association, is reported to have declared, on the authority of the Chairman of the Lords' Committee (Lord Sandhurst), that the Lords were not against the registration of nurses, and that our statement was in effect untrue. Dr. Bezley Thorne proceeded to say:—

> There would be hardly a member of the association who had not been made aware of a statement in the Press to the effect that the decided opinion of the Lords' Committee on Hospitals was adverse to the system of registration established by the association. There were few statements so absolutely fallacious that constant reiteration would not make them believed if people had not time to test their accuracy. Anyone who took up the third report of the Lords' Committee might ascertain for themselves that the Lords did not express any such opinion. There was interpolated in the report an observation by a witness or by a clerk who drew up the report, but when they looked to the part of the report containing the Lords' conclusions there was no statement about registration, and *it was, therefore, contrary to the truth to say that the Lords had expressed an adverse opinion as to the registration work of the association.*

In these circumstances it is interesting to note that in the full copy of the third report of the Lords' Committee on Metropolitan Hospitals, issued last week, precise evidence is forthcoming on the point in dispute. This report contains minutes of the proceedings at each sitting of the Committee, and also the full text of the Chairman's draft report. On pages cxcv. and cxcvi. the minutes of a meeting of this Committee, held on June 22nd last, are given. Paragraph 598 of the Report, as drafted by the Chairman, was as follows:—

> British Nurses' Association.
>
> Your Committee considered that the arguments in favour of the registration of nurses outweigh those against it, and they recommend that the charter desired by two associations should be granted.

Lord Sandhurst having moved the adoption of this paragraph, it was rejected by six votes to two. Lord Sandhurst and Lord Thring voted for its insertion, whilst Earl Cathcart, the Earl of Kimberley, Lord Zouche of Haryngworth, Lord Clifford of Chudleigh, Lord Sudeley, and Lord Monkswell voted against it.

The Lords' Committee, on a division, after hearing all the arguments for and against the proposals of the R.B.N.A. during sittings extending over nearly three years, deliberately determined, by six votes to two that the arguments in favour of the registration of nurses do not outweigh those against it, and that they could not, therefore, recommend that a charter should be granted to the associations seeking it.

Our readers will perceive that the conclusions we drew from paragraphs 478 to 483 of the Lords' Committee Report erred, if they erred at all, in taking too favourable a view of the action of the Committee of the House of Lords in regard to Nurse Registration.

The facts being as stated, we must express a hope that Dr. Bezley Thorne will, in all fairness, admit the mistake into which he has been led, no doubt inadvertently, and that he will make due apology.

### Training in Massage.

We have at the present day to face the fact that annually a large number of women who have enjoyed protection and home comforts from childhood are cast into the vortex of the unskilled who are struggling to obtain the means of subsistence. At an age when it appears to them futile to spend long years in an apprenticeship, and without the means of supporting themselves during such a time of training, they, nevertheless, find that they must turn their attention to some profession or trade that will give, in addition to daily bread, some appearance of the standing that has appertained to them in happier days. Hence it is in answer to the demand for quick training at a small cost that a number of persons are always to be found who profess to teach a skilled occupation in an incredibly short period.

It is far from the intention of the writer of this article to say that the above remarks apply in their entirety to massage. It may be possible to teach this delicate manipulative treatment, together with the amount of theoretical and scientific knowledge required, in the brief space of two or three weeks, but the fact remains that the public and many medical men are beginning to look askance at a calling that is learnt at so small an expenditure of time and money, and which yet professes to do so much for the patient. Two verdicts only are possible. Either that massage must be classed with those trades that require little skill and less experience, and so be paid accordingly, or it is a valuable aid to medical treatment and should be taught with the thoroughness and scientific accuracy that is employed in teaching other skilled occupations. The question to be answered is, Does massage simply consist of a series of movements which can be applied mechanically to any of the varied cases for which manipulative treatment is considered advisable? If the answer is in the affirmative, then those who undertake to teach the art in a few weeks are in the right. At the same time it is scarcely comprehensible how any treatment that is merely mechanical can be left to deal with the physiological processes which are set in action by either passive or active exercises. That individuals differ greatly in degree is exemplified by the well-known fact that one person will bruise or blister under a pressure that is scarcely felt by another, and if the comparatively hardened surface will show such different results, it is open to supposition that the delicate and complicated internal structures require to be treated with an amount of judgment and skill that is only gained by careful training and experience.

It has been said that the larger number of women who learn massage are nurses, and therefore previous training has rendered much theoretical teaching unnecessary, but it is open to doubt whether the trained nurse often settles down as a masseuse; usually she combines the two professions, and her massage cases being few and far between, she is but an indifferent operator. Besides, previous training in nursing, though extremely valuable, does not take the place of the special knowledge and practical acquaintance with manual exercises that combine to make a first-class masseuse.

It is curious to find with what gravity the Swedish professors regard the study of this treatment. Ling, the first great master of medical gymnastics, of which massage forms an important part, though not a medical man, "learned everything that could in those days be learned in the different departments of medical study." So important were these exercises considered that in 1888 it was decided by the heads of the College at Stockholm that students of Ling's system must pass a three years' course of study before obtaining a certificate of proficiency. The last year was spent in attending lectures on diseases, and in the practical application of various movements to patients undergoing treatment.

Though the therapeutic effect, and therefore the study of massage, is held of far less value here than in Sweden, there are one or two schools that train their pupils more carefully than the others, procuring for them the necessary advantage of lectures on physiology and anatomy. But these schools do not teach the art in less than three months, and the cost is, of course, in proportion. Hence fewer students attend the classes, though it is satisfactory to find that owing doubtless to the greater value of their certificates they have no difficulty in obtaining work. It would probably be beneficial if the course were lengthened to six months, and an effort made to obtain the clinical material, for want of which much is left to be learnt at the expense of future patients. The touch of an unpractised hand was once felt by the writer, and the remembrance is still bitter. The matter appears to require a thorough investigation, and if manipulative treatment is, as it is affirmed to be, a valuable addition to therapeutics, it should be taught and studied in a manner worthy of its beneficent qualities.

### The Workhouse Infirmary Nursing Association.

In noticing the past year's work, one cannot help thinking that there is no section of the nursing world which more deserves recognition and interest than that which has given itself to utilizing the enormous field for the systematic training of nurses which is present in our workhouse infirmaries; and what is of more consideration even than the opening of the new field of training is the alleviation of misery and unnecessary torment to sick people which is effected by making use of it. Inhuman guardians of the poor are becoming fewer in number every year, and if ratepayers have the satisfaction of knowing that their money is being used for a beneficent purpose, they owe that satisfaction in large measure to those who, with the courage of their opinions, have made such vast improvements in our poor law infirmaries.

It is with great pleasure that we hear of the strides that are being made in Halifax, where Miss Wilkie has gone to take charge of the nursing, and where we hear of such good progress in every direction. There is now a nursing home separate from the infirmary, cleanliness and order reign supreme, and a limited number of probationers are receiving training there. When we remember that it was only in August, 1891, that the committee appointed to consider the question of improved nursing visited and reported on the systems in used at Liverpool and Birmingham, it shows what can be done in a year, and reflects the greatest credit on those who took the work in hand.

From Norwich comes the good news that the guardians have consented to appoint a trained superintendent of nurses, and Miss Emma Maxwell, who has had six years' work at Whitechapel Infirmary, and who is a three years' Guy's certificated nurse and holder of the L.O.S. diploma, has been chosen to fill the post. She will have the assistance of four fully-trained nurses, and will begin work on November 25th.

In the recently-built infirmary at Reading there will be three trained assistant nurses under the head nurse, Nurse Pinnington, who has been working there since 1880.

One of the greatest points to gain is the appointment of the trained superintendent of nursing, for it is a significant fact that, whatever frictions and difficulties have ever occurred where trained nurses have been working have generally arisen in difficulties between them and an untrained master and mistress. All of us, including nurses, are only mortal, and we naturally more readily obey one who, by experience and education, is fitted to be our superior, than one who cannot judge of the efficiency of our work. The W.I.N. Association has compiled some sensible rules for nurses working single-handed, or at any rate with no trained superintendence; and it is satisfactory to know that several boards of guardians have adopted them. During the half-year ending last September, thirty-three nurses were appointed to posts by the Association, and twenty probationers have entered for training; and excellent as the extended work has been during the year, it is nothing to what might be done if more subscribers could be found towards the funds. Undoubtedly one of the hardest workers towards reform is the trained nurse who finds herself alone amongst untrained officials, for the old prejudice against "new-fangled notions" is not yet extinct, and to insist on the simplest sanitary measure may bring volumes of wrath on her unprotected head. But let such nurses work on steadily, comforted by the assurance that those of us who are really interested are mindful and grateful for her efforts, and that the slightest point gained in a whole day brings us one step nearer in the right direction.

### Appointments.

Oldham Infirmary.—Miss J. J. Hunter has been appointed Matron at this infirmary.

Indian Nursing Staff.—Miss Anne E. A. Hunt, has been appointed Sister in the Indian Nursing Service, and sails in H.M.S. *Serapis* on November 24th. Miss A. Hunt trained at St. George's Hospital and Rotunda Hospital, Dublin, and has been engaged in private nursing for the Co-operation of Nurses, 8, New Cavendish Street, Portland Place, W.

Eye and Ear Hospital, Tunbridge Wells.—Miss R. L. Harman Brown has been appointed Matron at this hospital, and has already entered on her duties there. Miss Brown was trained at the Royal Hospital, Portsmouth. She has since worked as a Queen's nurse at Cardiff, and as a district nurse at Harborne, Birmingham. She had the entire charge of Caterham Valley Cottage Hospital during the Matron's absence, and has excellent testimonials from all her appointments.

Guest Hospital, Dudley.—Miss Jessie Frances Parsons has been elected Matron of this hospital, and begins work there in December. Miss Parsons trained at the Wolverhampton General Hospital, and, after her three years' training, was in charge of one of the men's surgical wards there; she afterwards took a certificate at the York Road Lying-in Hospital, receiving her training there under Dr. Charles Cullingworth's supervision, and she also holds the L.O.S. diploma. Miss Parsons has also had work with the Bolton District Association, and acted as *locum* to the Matron at Weybridge Cottage Hospital. Her present post is that of Assistant Matron at the Derbyshire Royal Infirmary. Miss Parsons holds excellent testimonials, and is in every way fitted for the post to which she has been elected.

### Death in our Ranks.

On October 28th, amidst the deepest sorrow of all who knew her, Nurse Ingram passed away to her rest, after one week's intense suffering from diphtheria. She had just completed her third year of earnest work in the South-Western Hospital, Stockwell, and was greatly beloved and respected by all the members of the nursing staff. This sad event has cast a gloom over the entire hospital. Her age was 21.

On Tuesday, October 25th, Nurse Sparks, for three years nurse at Chichester Infirmary, and since 1885 parish nurse at Henley, passed away, beloved by rich and poor. She is buried in Henley Cemetery.

### Notes and Queries.

### Queries.

(24) *Rooms Wan'ed.*—Can anyone tell me of a nice place in the neighbourhood of Cavendish Square where anyone engaged in the nursing profession could stay during a visit to town?—*Inquirer.*

(25) *Reciprocal Term.*—Can anybody tell me whether it is possible to get experience in nursing on reciprocal terms for a few weeks, while waiting for a vacancy as probationer in a London Hospital?—*Inquirer Number Two.*

### Answers.

(23) *Male Nurses (T. J.).*—The article was written on all in general, and none in particular. Why single yourself out?

(19) *Books Wanted (Nurse F.).*—Dr. Willingham Gell's "Aids to the Sick and Injured," is published by the National Health Society, 53, Berners Street, W., in two forms—paper cover twopence, stiff boards sixpence.—*Nina.*

### CONSOLATION.

"Let not your hearts be troubled, believe in Me," were our Lord's words to His disciples just before the great trial of their faith, when His life was ended by a death of ignominy, and His followers, in fear and trembling, forsook Him and fled. He knew what they would suffer, and counselled them in their hour of darkness to trust in Him. Dismay fills our hearts when without warning we suddenly become the victims of a fell disease or a terrible accident. It is an awful trial to our faith; we know not what to do or to whom to fly for help. A thousand thoughts crowd our minds; uppermost are the questions, Shall I die? Am I fit to die? or, Shall I partially recover and be a miserable cripple for the rest of my days? We are overwhelmed by either alternative. The timid child in panic fear at some imaginary danger, rushes to its parent's side for protection, and, clasped tightly in loving arms, looks from its safe retreat with calmness on what had appeared so formidable. And shall not we fly to our loving Father for consolation in time of need? His Everlasting Arms will be our shelter, in them we may rest. God gives us strength, and all He asks of us at these times is, that we should learn to go forth and meet our future in the spirit of Him who to His prayer for deliverance added, "Not as I will, but as Thou wilt."

Then when the first anguish of our soul passes off, it will not leave only a dumb, dull resignation behind it, a feeling that "what can't be cured must be endured," but the gift of heavenly joy, the peace which passeth all understanding will steal into our hearts as soon as we can recognise the Saviour as our only and all sufficient portion. No words of man can satisfy and calm the blind soul groping for Light and Life, faith in and love to God alone are our true bliss and felicity. Fervent and constant should be our prayers to Him who is our Shelter from the wind, our Refuge from the storm, our Rock, our Tower, our Stronghold in adversity. Open our eyes, O Lord! that we may see Thee as Thou art! If anything will calm our fears, disperse our doubts, and soften our cares, it will be the simple childlike trust in God, which keeps our eyes raised to Him, looking to His help and not on our own peril. We shall feel

. . . In that hour
From out my sullen heart a power
Broke, like the rainbow from the shower,
To feel, although no tongue can prove
That every cloud, that spreads above
And veileth love, itself is love.

### Some Australian Experiences.

(*Continued from page* xxviii.,

As Ararat was my destination I did not stay long in Melbourne, but started off again in the train, and a very queer sort of place I found it. It was difficult to imagine that at one time it was densely populated by gold miners, for wherever you turned there were worked-out "claims." This was a large alluvial field, the ground is turned up and sifted in every direction; a few Chinamen (as usual) persevered in making a living, but the white men had departed to Ballarat to make enormous fortunes. Of course, mining still goes on there, but it seems that the days of the "Welcome Nugget" are over.

Most of the people round about Ararat are fruit growers, wine growers, &c.; there is a little quartz mining done, but very little compared with Ballarat.

I went out to one of the vineyards, and tasted grapes till I could not distinguish one flavour from another; they all seemed alike to me; but the Frenchman who took us round, went to a deal of trouble in explaining the difference in each of the rows of vines.

I visited a fruit garden, which was a most wonderful sight; there were raspberries, strawberries, gooseberries, mulberries, blackberries, peaches, apricots, quinces, apples, pears, oranges, lemons, and pomegranates, all ripe together. This seems almost incredible, but it is quite true; the currants and cherries were just over. As to tomatos, they were growing in every direction, and I also saw some of the finest roses I have ever seen, it was all like a fairy tale to me.

I went up Mount Ararat; there is quite a dent on the top, "where the Ark rested," so they tell you.

I also visited the hospital, a very nice one it was too.

We paid a short visit to Ballarat, and found it a very delightful place, lovely public gardens with ornamental water; the whole place in fact has a very prosperous air about it.

I then returned to Melbourne and commenced work. I lived in a house where several other nurses resided, but was not at all prepossessed by their style. Miss McCartney, in East Melbourne, has since then opened a nursing institution, and this, no doubt, has raised the standard very considerably. Somehow I never cared so much for Melbourne, and was very glad to have got a case which lasted some time. One cannot help admiring it, it is such a wonderfully beautiful place for its age. The public buildings are magnificent, and the Fine Art Gallery, the Public Library, the Town Hall, the General Post Office, and the Bank are all very fine, and so are the Universities. There are several hospitals—the Homœopathic, the Women's, the Alfred, the Children's, and there are also several nursing homes. Mr. Fitzgerald, the Melbourne surgeon, has the largest of them.

Not only is the city well laid out, but the suburbs are very lovely, and the climate suits the English elms, limes, May trees, lilacs, and laburnums, and the native foliage being also very fine adds to the beauty of it all.

### Queensland.

At about this time I applied for and obtained the post of Superintendent of the Children's Hospital, Brisbane, so I proceeded thither by steamer. It was a very delightful trip—like a millpond all the way.

The change from Melbourne to Brisbane was great indeed; the latter is so very tropical, and its wooden houses on piles, with verandahs all round, surrounded by an abundance of flowering shrubs, creepers, and banana trees, which give the place a totally different aspect to either Sydney or Melbourne; in fact, I never really felt in the Colonies until I took up my abode in Brisbane. The external appearance of the Children's Hospital is exactly like "Noah's Ark"—it has a verandah round, and a great many doors and windows. Internally, it is a very comfortable place; it is two storeys high, the upper part containing the principal wards, the general dining-room, and the sitting-room and bed-room of the Lady Superintendent.

(*To be continued.*)

### Examination Questions.

ON CERTAIN POINTS IN THE NURSING OF SICK CHILDREN.

We received only fourteen answers to the question set in October, and the best of them was sent by Nurse Dora, Blaenavon, and is printed below. Many sent very long answers, whereas we wish them to be as concise as is compatible with the subject on which they are written. Several nurses forgot that observation on the part of a child's nurse is of immense importance, and cleanliness in the care of all utensils used for a child's food was another point missed over by one or two. We hope that our competitors, if not able to answer the questions from their own knowledge and experience, will read up the subject, and then having put away the books will proceed to write the reply, as, of course, if the answers are simply taken straight out of a hand-book no benefit is gained in the slightest degree. The question for November is: (1) What special portion of the body does the typhoid poison attack? (2) Why is it essential that typhoid cases more strictly than other fever cases should be kept in the recumbent position? (3) What symptoms would indicate internal hæmorrhage? Answers must be sent in by November 30th. Each answer must be accompanied by writer's name and address, must be short and concise, written on one side of the paper only, and addressed "*Nursing*" Editor of The Hospital.

### Prize Answer.

Sick children differ from adults in many ways; their appetites are more capricious, tempers more fretful, and they are more susceptible to rising of temperature from the slightest reason; they frequently cannot localise pain correctly, and demand more careful observation, tact, and intelligence than the adult. No one, even with the best intentions, can be a successful children's nurse without having a love for them. Patient, constant watchfulness, gentle firmness, cheerfulness, and a thorough knowledge of infants' dietary and general hygienic arrangements are some of the required principles. An exclusive milk diet is best until the first or milk teeth appear; then, whichever of the foods suit best may be used, but whatever it be, regularity must be observed; the feeding must not be hurried over, and if a bottle be used it should be kept scrupulously clean. The clothing must be light as well as warm—a flannel night-gown is best; nothing tight must be worn, and all soiled linen must be removed at once. In bathing, the water must be a proper heat—about the temperature of the body. Anything likely to frighten the child must be avoided; it should be done as gently, quickly, and thoroughly as possible, the drying very carefully attended to, particularly armpits, groins, and buttocks, which parts may be dusted over with starch, violet powder. or Fuller's earth powdered.

### Where to Go.

On Saturday, November 26th, at half-past seven, Mr. Walter F. Rawles will give a grand musical and dramatic performance in aid of the Convalescent Home Fund of Charing Cross Hospital. Besides the musical part of the programme, Tom Taylor's "Plot and Passion" will be played. Tickets, which are procurable at Charing Cross Hospital, will be from 1s. to 7s. 6d. each. After the last concert in aid of the same hospital, which Mr. Rawles got up, the sum of eighty pounds was handed over, and we wish a similar success may attend this undertaking.

## Figures and Tables

**Figure f1:**